# Job quality and employee happiness: evidence from China

**DOI:** 10.3389/fpsyg.2025.1550200

**Published:** 2026-01-07

**Authors:** Bingbing Yu, Zhenping Song, Zheng Shen

**Affiliations:** 1 School of Information Engineering, Hangzhou Dianzi University, Hangzhou, China; 2 Key Cultivating Think Tank, Hangzhou, China

**Keywords:** job quality, employee, happiness, subjective well-being, work

## Abstract

**Background:**

Despite the rapid economic growth experienced by China in recent decades, issues such as income inequality, occupational stress, and disparities in career advancement opportunities have become increasingly salient, exerting a detrimental influence on the subjective well-being and overall quality of life of employees.

**Methods:**

Using a nationally-representative survey data in China, we construct an index of job quality at the individual level using a multidimensional approach and then estimate the effect of job quality on employee happiness.

**Results:**

Results from instrumental variable estimation show that job quality has a significantly positive effect on employee happiness. Moreover, a high-quality jobs enhances employee subjective well-being mainly through pathways including improved mental health and increased harmony perception.

**Conclusion:**

To improve the well-being of employees in developing countries, this paper emphasizes the importance of policy support to improve the quality of their work.

## 1 Introduction

In many developing countries, large numbers of people are employed in low-quality jobs and face inequalities at work, which has profound implications on their subjective well-being. This phenomenon is of particular concern because job quality is as important as participation in work itself (Diener et al., 2018; Helliwell et al., 2022). For employees themselves, simply having a job does not guarantee a higher level of subjective well-being. For instance, in rapidly developing countries such as China, where industrialization and urbanization have increased employment opportunities, employees still often face physical and mental health problems and low occupational identity. These problems directly affect their happiness and satisfaction. Existing research has shown that factors such as poverty, health, and social capital do indeed affect an individual’s subjective well-being (Herman et al., 2013; Puntscher et al., 2015; Caria and Falco, 2018). However, studies on the relationship between job quality and employee happiness are less well understood. Particularly in developing countries, labor market segmentation leads to different levels of job quality and treatment, further exacerbating inequality in work (Van den Broeck and Maertens, 2017; Markussen et al., 2018). Therefore, understanding and enhancing job quality has become a critical component in advancing the Sustainable Development Goal of promoting decent work (United Nations, 2022).

The purpose of this paper is to examine the impact of job quality on employee happiness in the context of China. By constructing a multidimensional job quality index, this study considers four aspects, including employee rewards, employment security, employment conditions, and employment skills, which are key factors in measuring the job quality of Chinese employees. Based on the job demands-resources theory (Demerouti et al., 2001), we developed a theoretical framework to analyze the effect of job quality on happiness. To gain deeper insights into this relationship, we include improvements in physical health, mental health, fairness perception, and harmony perception as mechanism variables for analysis. Using data from 2020 and 2022 waves of the China Family Panel Studies and the instrumental variable approach, our analysis not only provides theoretical insights for understanding how job quality affects employee happiness, but also provides practical guidance for policymakers aiming to improve the job quality and subjective well-being of employees in developing countries.

The remainder of this paper is organized as follows. Section 2 presents the conceptual framework. Section 3 describes the data, variables, and summary statistics, and section 4 describes the empirical strategy. Section 5 presents the main results from regression models, robustness checks, and estimation results for the underlying mechanisms. In Section 6, we discuss the implications and limitations.

## 2 Conceptual framework

Job quality or the quality of employment has been studied in both low- and high-income countries, and significant advances in concept and measurement have been made in previous research. (Horowitz, 2016; Leschke and Watt, 2014; Van Aerden et al., 2016; Sehnbruch et al., 2020). Job quality is widely considered to be a multidimensional concept, a comprehensive reflection of an employee’s work situation and the fundamental human right to life, health and well-being, as well as the capabilities that should be available to ensure normal human participation in socioeconomic activities (González et al., 2021). Although scholars do not always use the same dimensions to evaluate the quality of employment in their analyses due to the availability and content of suitable datasets, most scholars consider job quality to include a combination of the aspects related to rewards, job security, working conditions, and employment skills.

Based on job demands-resources (JD-R) theory, we construct a conceptual framework to discuss how job quality affects personal happiness. The JD-R theory argues that health and self-perception are two key factors influencing one’s subjective well-being. Given the multidimensional nature of job quality, we propose that job quality can affect employees’ happiness through two main channels: the first is the health status channel, and the other channel is through self-perception.

We constructed a more comprehensive job quality index based on the JD-R theory and explore the impact of job quality on employee happiness. When discussing the impact of job quality on employee happiness, the JD-R theory provides an important framework for understanding the relationship between job quality and employee happiness. This theory argues that any job characteristic can be divided into job demands and job resources. Job demands refer to the demanding requirements in work that consume employees’ psychological, physiological, and social resources, such as work pressure, time urgency, and role conflict, etc., these factors will reduce health and energy, leading to serious mental disorders over a period of time (Demerouti and Bakker, 2011). According to the health impairment process, high job demands requiring continuous effort may deplete employees’ resources, leading to burnout and health issues (Caplan et al., 1975). Specific job demands (e.g., work pressure or emotional demands) have repeatedly been found to predict exhaustion (i.e., severe fatigue) across various occupational groups (Bakker et al., 2005; Bakker et al., 2003; Demerouti et al., 2004), while occupational burnout has a significant negative impact on employees’ happiness (Adil and Baig, 2018). Job resources refer to positive factors that help employees achieve work goals, alleviate work pressure, and promote personal growth, such as job autonomy, social support, and performance feedback, etc., these factors can motivate employees and mitigate the impact of higher job demands (Demerouti and Bakker, 2011). Furthermore, according to the motivational process proposed by the JD-R model, job resources, due to their motivational potential, encourage employees to achieve their goals (Demerouti et al., 2001). In turn, employees may become more engaged in their work because they gain a sense of achievement from it (Hackman and Oldham, 1980), thereby further enhancing happiness. Therefore, according to the Job Demands-Resources theory, the impact of job quality on employee happiness can be explained by balancing the dynamic relationship between job resources and job demands. The job quality index constructed in this article covers four dimensions—employee returns, employment security, employment conditions, and employment skills, which, respectively, correspond to core resources and demands in the JD-R theory. They collectively influence employees’ health and psychological state, ultimately impacting their happiness.

Given the multidimensional nature, the job quality can affect employee happiness through two major channels. The first channel is the health channel. That is, good-quality jobs are associated with better workplace environments and health behaviors, which have positive effects on happiness. On the one hand, A good workplace allows employees to engage more in health-promoting behaviors and reduces job burnout, thereby improving their physical and mental health and enhancing employee well-being. Such an environment not only promotes an active lifestyle among employees but also decreases psychological and physiological issues caused by work stress, thus increasing overall job satisfaction and quality of life. On the other hand, socioeconomic disparities in health behaviors suggest that people in higher socioeconomic status more often act in ways that benefit their health than those in lower socioeconomic status (Pampel et al., 2010). The working conditions of higher socioeconomic groups contribute to the development of higher levels of self-efficacy (Bandura, 1977), and it may promote personal health status. Furthermore, the threat of job insecurity is likely to increase occupational stress, anxiety, and depression, which negatively affect the emotional and psychological well-being of workers.

The other channel that the job quality impacts happiness is through self-perception. The enhancement of job quality and the associated changes in self-perception may also affect a person’s happiness. Previous studies have shown that working does not simply function as a means of earning a livelihood, but more importantly achieve a positive feeling and expectation fulfillment; working means having a fair chance in life, gaining respect, and improving social status (Morse and Weiss, 1955; Mor-Barak, 1995). However, the self-perception for employed workers can be higher or lower, which depends in part on job characteristics such as stress and prestige as well as the social, physical and policy environment factors. Decent jobs that pay well and offer work-life and gender balance can lead to a higher degree of self-esteem and an increased sense of accomplishment, which is positively associated with subjective well-being. On the contrary, less positive self-perceptions and greater distress are more prevalent among workers with low-skill work, temporary jobs, and lower job status (Kelloway and Barling, 1991; Link et al., 1993).

## 3 Data and variables

### 3.1 Data

The data for this analysis are obtained from the 2020 and 2022 waves of the China Family Panel Studies (CFPS), which is conducted by the Institute of Social Science Survey (ISSS) at Peking University. The CFPS uses a multi-stage probability strategy to draw data on a target sample size of 16,000 households from 25 provinces in China. It contains a wide range of data on individual’s labor market, such as employment status, job sector, and work conditions, and a variety of survey questions about job characteristics for working people. Since this article investigates the link between the job quality and employee happiness, our analysis is confined to adult men and women whose primary job is wage employment. The final sample for the main analysis consists of 15,013 individuals, after adjusting for missing values and invalid responses in all variables used in the study.

### 3.2 Employee happiness

In our study, happiness is measured by the answer on the question, “Overall, do you think you are happy with your life?” Respondents are able to choose from a 10-point scale, ranging from “1 = very unhappy” to “10 = very happy.” An advantage for this question is that respondents can understand more easily.

### 3.3 Job quality

We construct an index of job quality based on the Alkire and Foster (AF) method that uses a dual cut-off approach to measure multidimensional poverty (Alkire and Foster, 2011; Sehnbruch et al., 2020). The job quality index used in this study is composed of four dimensions and 12 indicators (Table 1). While the variables included in this index are not exhaustive due to data limitations, such a technique that simultaneously considers multiple features of job quality can illustrate the extent to which employees are achieving essential capabilities and functioning in their respective labor markets (González et al., 2021).

**Table 1:** Dimensions, indicators, and weights.

Dimension	Indicator	Deprivation cut-off	Weight
1. Employee rewards	(1) Wage incomes	Below the 25% quantile of the income distribution	1/8
	(2) Non-wage benefits	There is neither a housing allowance nor a meal allowance	1/8
2. Employment security	(1) Social insurance	Do not have any of the five types of social insurance	1/16
	(2) Housing Provident Fund	Do not participate in the Housing Provident Fund scheme	1/16
	(3) Labor contract	Had never signed a formal labor contract with the employer	1/16
	(4) Union membership	Not a member of a trade union	1/16
3.Employment conditions	(1) Working hours	Works more than 40 h per week	1/16
	(2) Occupational safety	Being dissatisfied or very dissatisfied with safety in the job	1/16
	(3) Workplace stability	Employees work primarily outdoors	1/16
	(4) Autonomy	Employees work exclusively on a fixed commute schedule	1/16
4. Employment skills	(1) Foreign language use	Does not use foreign languages at work	1/8
	(2) Computer use	Does not use computers at work	1/8

#### 3.3.1 Employee rewards

The reward of work is not just a crucial economic resource, but it is also a summary measure of the value and performance of a worker in the labor market. Following Van Aerden et al. (2016), the first dimension considers the employee rewards, which includes wage incomes and non-wage benefits. The 25% quantile of the income distribution is defined as a deprivation cut-off for wage incomes. The logic behind this definition is that using a relative income indicator could reveal people’s utilities which depend largely on their own income compared with that of others (Shen et al., 2022). Non-wage benefits are defined as employees who are deprived if the employer does not provide either a housing allowance or a meal allowance.

#### 3.3.2 Employment security

This dimension includes social insurance, Housing Provident Fund, labor contract, and union membership. Social insurance defines employees as deprived if they do not have any of the five types of social insurance. In China, enterprises are required to pay five social insurance contributions for their employees: workers’ pension, workers’ medical insurance, work injury insurance, unemployment insurance, and maternity insurance. Housing Provident Fund considers employees to be deprived if they do not participate in the Housing Provident Fund scheme, as it plays a necessary role in ensuring the housing affordability for workers to reduce the pressure of housing costs on their lives (Zhan et al., 2022). Labor contract defines employees as deprived if they had never signed a formal labor contract with the employer. Union membership has the potential to provide formal support for the enhancement of an individual’s social capital, which is a primary source of subjective well-being (Shen et al., 2022). Employees who are not belong to the membership of a trade union at the workplace are defined as deprived in this indicator.

#### 3.3.3 Employment conditions

Following the work of Organisation for Economic Co-operation and Development (OECD), 2014, employment conditions include four indicators: working hours, occupational safety, workplace stability, and autonomy. The working hours indicator is derived from the number of hours an employee works per week, and an employee is considered to be deprived if they work more than 40 hours per week (i.e. long working hours). Past research has shown that long working hours are significantly associated with poor health and are a cause of lower work-life satisfaction (Wu et al., 2019; Sato et al., 2020). Occupational safety considers employees to be deprived if they are dissatisfied or very dissatisfied with the safety in their current jobs. Workplace stability represents the stability associated with work environment, and it is deprived if employees work primarily outdoors. Autonomy measures the importance of having a job with work-life balance, and it considers employees to be deprived if they must work exclusively on a fixed commute schedule.

#### 3.3.4 Employment skills

Employment skills should be considered as an important feature of the job quality from the perspective of career development, as it plays an essential role in the promotion of an employee human capital accumulation in the context of work (Van Aerden et al., 2016). This dimension comprises the indicators of foreign language use and computer use. The former considers employees to be deprived if they do not receive any form of foreign language use, and the latter considers that employees are deprived if they have never used a computer at work.

### 3.4 Control variables

We controlled for a set of variables related to individual and household characteristics that are both correlated with job quality and happiness. Individual characteristics include age, gender (1 = female, 0 = male), marital status (1 = being married, 0 = otherwise), education (years of formal schooling completed), religious beliefs, and ethnic identity. Household characteristics include household debt (equal to 1 = household in debt, 0 = otherwise) and the number of children in the family (aged 6 or younger, aged 7 to 12, and aged 13 to 18). Furthermore, we include dummy variables for manufacturing, construction, transportation, and services. Additionally, we control for the number of junior high schools, the number of high schools, and the number of universities in the respondent’s province to account for regional educational resources.

## 4 Empirical strategy

### 4.1 The basic model

Formally, a linear regression model is used as the basic mode to estimate the overall effect of job quality on employee happiness:


$$Y_{\mathrm{ijt}}=\beta_{0}+\beta_{1}\text{qualit}y_{\mathrm{ijt}}+X\prime \phi +\theta D_{t}+\delta C_{j}+\mu_{\mathrm{ijt}}$$ (1)

Where $Y_{\mathrm{ijt}}$ denotes the happiness for the individual i living in county j in year t . $\text{qualit}y_{\mathrm{ijt}}$ is the independent variable of primary interest, an index measuring the job quality for each employee. X is a vector of control variables reflecting the characteristics of individual, household, and sector. $D_{t}$ and $C_{j}$ are dummy variables to account for year and county fixed effects. Robust standard errors are used in all regression models. We are particularly interested in the coefficient $\beta_{1}$ , which reveals the effect of job quality on employee happiness. When estimating the Equation 1 with ordinal outcomes, we focus our attention on a linear regression model for estimation to accommodate the linear instrumental-variables model in the presence of endogeneity discussed in the following section. Linear regression models are commonly used for estimation because of their simplicity and proper approximation (Shen et al., 2023), and it has been shown that using ordered outcomes as the dependent variable yields very similar estimates (Ferrer-i-Crabonell and Frijters, 2004; Aldén et al., 2020).

### 4.2 Endogeneity

Our results may still be biased due to the issue of endogeneity. One concern for identifying the causal effect is the reverse causality problem. For instance, happier people tend to have better social relationships and are more likely to have access to good-quality jobs, which in turn could lead to high levels of subjective happiness. The other concern is omitted variable bias. Unobservable factors such as an individual’s biological endowment or tastes for work are likely to be associated with both job quality and subjective well-being. These two scenarios would result in an overestimation or underestimation of job quality effect on one’s level of happiness or satisfaction. We address the endogeneity by applying an instrumental variable (IV) approach to our data. Specifically, we need to find an exogenous variable to instrument for the $\text{qualit}y_{\mathrm{ijt}}$ which does not impact $Y_{\mathrm{ijt}}$ directly, but through its effect on $\text{qualit}y_{\mathrm{ijt}}$ . Therefore, the first stage equation of the two-stage least-squares (2SLS) technique is defined as follows:


$$\text{qualit}y_{\mathrm{ijt}}=\alpha_{0}+\alpha_{1}Z_{\mathrm{ijt}}^{1}+\alpha_{2}Z_{\mathrm{ijt}}^{2}+X\prime \varphi +\lambda D_{t}+\omega C_{j}+\varepsilon_{\mathrm{ijt}}$$ (2)

Where $Z_{\mathrm{ijt}}^{1}$ and $Z_{\mathrm{ijt}}^{2}$ are instrumental variables for job quality, and other variables are the same as described in Equation 1. The first instrument $Z_{\mathrm{ijt}}^{1}$ represents an individual’s level of Mandarin proficiency, which is measured by the interviewer to record whether the respondent used Mandarin during the interview. Mandarin proficiency should be relevant because proficiency in the standard official language is considered to be an important form of human capital that is strongly associated with one’s labor market outcomes. The reason for this is that there is a positive correlation between national language skills and social network, use of modern production technologies and labor productivity (Gao and Smyth, 2011; Liu et al., 2020). The use of Mandarin helps to reduce the communication barrier and serves as a signal of one’s ability, which can increase the access to the high-quality of employment opportunities (Xu and Liu, 2023).

The second instrument $Z_{\mathrm{ijt}}^{2}$ represents an interviewer’s subjective evaluation of the respondent’s intelligence level, which is assessed objectively by the interviewer rather than relying on the respondent’s subjective reports. This evaluation is based on a scale ranging from 1 to 7, where 1 indicates very low intelligence and 7 indicates very high intelligence. The interviewer evaluates the respondent’s intelligence level through direct interaction and observation during the interview process. This instrument should be relevant as there is a high correlation between intelligence levels and job quality. Employers are more likely to hire individuals with higher intelligence and offer them positions that demand higher performance and quality of work. Intelligence level reflects an individual’s intrinsic cognitive abilities, which are directly related to job performance and the quality of employment. Higher intelligence can enhance human capital and contribute to better educational attainment and employment opportunities in adulthood (Hanushek and Kimko, 2000; Murphy and Peltzman, 2004). Thus, this measure of intelligence serves as a robust predictor of future employment outcomes and job quality.

While the relevance of the instruments can be easily tested using the first-stage regression results, the exogeneity of the instruments is needed for more description and explanation. We argue that the instruments are plausibly exogenous. For the first instrument, Mandarin proficiency should be exogenous with respect to respondents’ happiness, as the level of respondents’ Mandarin ability is assessed by trained interviewers rather than self-reported by participants. Therefore, Mandarin proficiency measured by an objective indicator appears to be independent of employees’ well-being outcomes and does not directly affect their own feelings and emotional reactions.

For the second instrument, the objectively evaluated intelligence level reflects the employee’s cognitive abilities and resilience, which are not influenced by his or her psychological status in adulthood. Specifically, the respondent’s intelligence level is assessed by trained interviewers rather than self-reported by the participants. Therefore, intelligence measured by objective indicators appears independent of the employee’s well-being. Also, the impact of personality traits in adolescence on the level of well-being in adulthood is not direct, as it is likely to be mediated through current employment status and working conditions. This objective assessment of intelligence provides a more accurate reflection of an individual’s cognitive abilities without being influenced by their current psychological state or subjective self-assessment. Consequently, it serves as a robust predictor for future employment outcomes and job quality.

However, we acknowledge that results from IV estimation might be sensitive to the exclusion restriction; that is, potential factors associated with instruments could influence individual happiness. Respondents’ well-being in adulthood is likely influenced not only by their experience of migration but also by their family’s socioeconomic status during adolescence. These factors could pose threats to the validity of our IV estimates if they are correlated with both the instrument and the outcome, thereby violating the exclusion restriction.

To address these potential threats, we have included two variables in the IV regression model to control for early-life experiences: interviewers’ migration experience since the age of 14 (1 = yes, 0 = no), and family’s social class at the age of 14 (“1 = bottom rung” to “5 = top rung”). While these controls aim to mitigate some sources of endogeneity, it is important to recognize their limitations. For instance, interviewer’s migration experience might not fully capture the complexity of migration impacts on an individual’s subjective well-being. Additionally, categorizing family social class may oversimplify the nuanced influences of socioeconomic background on adult outcomes.

## 5 Results

### 5.1 Summary statistics

Table 2 presents summary statistics of variables for this analysis. The mean values of happiness is 7.408, which is above the midpoint of 5 (Neither happy nor unhappy). The average of job quality index is slightly below 0.5, implying that most of employees in the sample are associated with comparatively low quality jobs. The average age is about 42 years, and 56 and 85.3% of the respondents are female and married, respectively. The proportion of respondents from an ethnic minority group is 5.8, and 1.8% of people have a religious belief. About 2.7% of the respondents reported that their households were in debt. The means for the number of children of different ages in the household are quite similar.

**Table 2:** Summary statistics of main variables.

Variables	Mean	Standard deviation
Dependent variables		
Happiness	7.408	1.979
Key independent variable		
Job quality	0.474	0.206
Control variables		
Age	42.03	11.48
Female	0.560	0.496
Married	0.853	0.354
Education	10.86	4.144
Religious beliefs	0.018	0.134
Ethnic minority	0.058	0.234
Household debt	0.027	0.161
Number of children aged 6 or younger	0.297	0.571
Number of children aged 7 to 12	0.281	0.561
Number of children aged 13 to 18	0.202	0.466
Manufacturing	0.256	0.436
Construction	0.115	0.319
Transportation	0.048	0.214
Services	0.329	0.470
Other sectors (reference)	0.198	0.399
Number of universities within the province	111.0	38.18
Number of high schools within the province	619.6	262.6
Number of junior high schools within the province	2,381	1,255

### 5.2 Estimation results for job quality and happiness

Table 3 presents the estimation results using OLS models that assume the job quality to be exogenous, as well as the results from IV models with Mandarin proficiency and intelligence level as instruments for job quality. In all regressions, we control for individual, household, and sector characteristics, and year and county fixed effects. In column (1) that presents the OLS regression for happiness, job quality is significantly and positively associated with employee happiness. This suggests a higher level of happiness for people with better quality jobs than that of those employed in poorer quality jobs. We estimate the impact on happiness by using an instrumental variable procedure, as displayed in columns (2)–(4). Estimation results based on only one instrumental variable, Mandarin proficiency or intelligence level, are presented in columns (2) and (3), while results from the regression model using two instruments simultaneously are presented in column (4). Overall, we find that the IV estimates are positive and significant.

**Table 3:** Estimation results for job quality and happiness.

	OLS	IV	IV	IV
	(1)	(2)	(3)	(4)
Job quality	0.412***	2.628	14.163***	9.363***
	(0.100)	(3.158)	(3.960)	(2.545)
Individual-level controls	Yes	Yes	Yes	Yes
Household-level controls	Yes	Yes	Yes	Yes
Sector controls	Yes	Yes	Yes	Yes
Province-level controls	Yes	Yes	Yes	Yes
Year FE	Yes	Yes	Yes	Yes
County FE	Yes	Yes	Yes	Yes
First-stage coefficients: Mandarin proficiency		0.016*** (0.005)		0.016*** (0.005)
First-stage coefficients: Intelligence level			0.005*** (0.001)	0.005*** (0.001)
First stage F-statistic		10.1	15.62	12.43
Observations	15,013	15,013	15,013	15,013

***, ** and * indicate significance at the 1, 5 and 10% levels, respectively. Robust standard errors are reported in parentheses.

The validity of an instrument relies on the assumption of relevance and exogeneity. We first examine the relevance of the instruments. Table 4 also reports first stage results of the IV models. We find that our first stage relationship is strong and results are robust to the specifications with adjustments for different instrumental variables. Specifically, the association between Mandarin proficiency and job quality is significant and positive, which is related to a high likelihood of access to good quality jobs for employees if they are considered fluent in Mandarin. Intelligence level is a strong predictor for the job quality among employees, with an estimated coefficient that is positive and significant at the 1 % level. Furthermore, the values of the first stage F-statistic for the weak identification are above 10 across all specifications, indicating that the chosen instruments are not weak and sufficiently explain the variation in job quality.

**Table 4:** IV regression models with additional controls.

	Happiness		
	(1)	(2)	(3)
Job quality	9.016*** (2.966)	9.404*** (2.958)	9.403*** (2.981)
Migration experience	−0.291* (0.174)		−0.309* (0.176)
Family’s social class		0.282*** (0.029)	0.282*** (0.029)
Individual-level controls	Yes	Yes	Yes
Household-level controls	Yes	Yes	Yes
Sector controls	Yes	Yes	Yes
Province-level controls	Yes	Yes	Yes
Year FE	Yes	Yes	Yes
County FE	Yes	Yes	Yes
First stage F-statistic	8.0	8.2	8.1
Observations	8165	8165	8165

***, ** and * indicate significance at the 1, 5 and 10% levels, respectively. Robust standard errors are reported in parentheses.

We next consider the exclusion restriction of instrumental variables. To ensure that no other variable explains the impact of Mandarin proficiency and intelligence level on happiness other than job quality, we include several controls that may be correlated not only with the instruments but also with the outcomes of interest. Estimation results for the IV models accounting for the potential factors (i.e., migration experience since the age of 14, and family’s social class at the age of 14) are reported in Table 4. The pattern does not change in all models where the estimates of job quality remain positive and significant. These results suggest that, migration experience and family characteristics are not the key channels through which the instruments influence an individual’s happiness. Again, the relevance of the instruments are not violated, as indicated by the value of the first stage F-statistic for weak identification.

### 5.3 Underlying mechanisms

Based on the mechanism discussed before, we investigate the potential channels of health and self-perception in this section. Note that for each mechanism, we use an OLS model as the baseline analysis and an IV model to address the endogeneity of the employment quality. Here, we begin by examining the health channel through estimating the impacts of job quality on physical health improvement and depressive symptoms. The physical health variable is indicated by self-reported health evaluation: 1 = worse health, 2 = unchanged health, 3 = better health. The mental health is indicated by depressive symptoms variable, aggregated from responses to questions such as feeling down, finding everything effortful, poor sleep quality, feeling cheerful, feeling lonely, enjoying life, feeling sad, and feeling that life is not worth living. Higher scores indicate poorer mental health among employed workers. Panel A of Table 5 shows the estimation results of the impact of job quality on physical and depressive symptoms. Regarding physical health, while OLS estimates show a marginally significant relationship between job quality and employees’ physical health, IV estimates suggest no significant causal effect of job quality on physical health improvement after accounting for potential endogeneity. This may be attributed to the context of China’s labor market where, despite higher subjective well-being due to improved job quality, intense work competition could lead to scenarios like increased employee rewards but deteriorating physical conditions, indicating a partial conflict between JD-R theory and reality in the unique context of rapid economic transition in China. Concerning mental health, both OLS and IV estimates indicate that high-quality employment significantly reduces depressive symptoms among employed workers, thus positively impacting mental health.

**Table 5:** Mechanism analysis.

	OLS	IV	OLS	IV
Panel A: Health	Physical health status		Depressive symptoms	
	(1)	(2)	(3)	(4)
Job quality	0.061* (0.035)	−0.089 (0.646)	−0.497** (0.237)	−7.878*(4.566)
First stage F-statistic		13.26		13.26
Observations	10,098	10,098	10,098	10,098
Panel B: Self-perception	Fairness perception		Harmony perception	
	(5)	(6)	(7)	(8)
Job quality	0.201*** (0.052)	0.337 (0.974)	0.130** (0.052)	3.113*** (1.132)
First stage F-statistic		13.26		13.26
Observations	10,098	10,098	10,098	10,098

***, ** and * indicate significance at the 1, 5 and 10% levels, respectively. Robust standard errors are reported in parentheses. All regression models control for individual, household, and sector characteristics, as well as year and county fixed effects. Additionally, provincial-level control variables, such as the number of junior high schools, the number of high schools, and the number of universities, are included.

We further investigate the self-perception channel, which includes individuals’ perception of fairness and harmony. The fairness perception variable is represented by respondents’ evaluation of having “a great opportunity to improve living standards.” The harmony perception variable is represented by respondents’ agreement with the statement “fair competition leads to harmonious interpersonal relationships,” with higher values indicating greater agreement. Estimation results are shown in Panel B of Table 5. Although coefficients from the OLS models are positive and statistically significant, the regression coefficient of the fairness perception variable on job quality from the IV model is not statistically significant. This might be due to excessive labor supply in the context of China’s rapid socioeconomic transformation, potentially weakening the association between our instruments and the endogenous variables. OLS estimates indicate a significant positive impact of job quality on perceptions of fairness and harmony, and IV estimates confirm a significant positive impact of job quality on harmony perception. Furthermore, the first-stage F statistic supports the validity of the instruments. These findings regarding self-perception suggest that high-quality jobs contribute to enhancing employees’ harmony perception, thereby improving their happiness.

## 6 Conclusion and discussion

This paper presents an empirical analysis for the impacts of job quality on employee happiness using micro-level survey data from China. An instrumental variable approach is applied to correct for the endogeneity bias and to infer the causal relationship. Our results show that employees with a high quality job have higher level of happiness. Furthermore, we analyze how job quality affects employees’ happiness by improving their health (physical health status and depressive symptoms) and self-perception (fairness perception and harmony perception). To help policymakers and businesses better implement our findings, we offer several policy recommendations:

To improve employee health behaviors and self-perception behaviors by enhancing job quality, thereby increasing subjective well-being, governments and enterprises can adopt a series of comprehensive measures. In terms of salary income, the government should formulate or adjust minimum wage standards to ensure all workers receive a decent living standard, while encouraging enterprises to implement performance bonus systems based on employees’ work performance. Additionally, promoting non-wage benefit programs such as providing housing allowances or meal subsidies, particularly for low-income families, helps alleviate economic pressures and improves quality of life. Within employment security, perfecting the social security system ensures every worker enjoys basic social insurance coverage including pensions, medical care, unemployment, work injury, and maternity insurance, and strengthening supervision to ensure enterprises pay social insurance contributions on time and in full; enacting relevant laws and regulations requiring enterprises to sign formal labor contracts with employees and regulate contract content, protecting workers’ legal rights and interests, and intensifying supervision over the implementation of labor contracts to prevent illegal dismissals; encouraging and supporting the establishment of trade unions within enterprises provides a platform for employees to express opinions and demands, enhancing employees’ social capital and collective bargaining power through legislative protection of union members’ rights. Regarding employment conditions, the government should establish strict regulations against unnecessary overtime, ensuring weekly working hours do not exceed statutory limits and providing sufficient rest time for workers, and require corresponding compensatory measures from enterprises for mandatory overtime; promulgating occupational safety and health regulations mandates regular workplace safety inspections to ensure no safety hazards exist, and requiring employees in high-risk positions to complete necessary safety training before taking up posts to ensure each employee possesses adequate safety awareness and emergency handling capabilities; encouraging enterprises to grant more autonomy to employees allows them flexible task scheduling and timing, which helps reduce occupational stress and enhance mental health. Concerning employment skills, the government should increase investment in vocational education, especially vocational skill training for vulnerable groups, helping them acquire necessary skills to enter the labor market, encourage enterprises to provide on-the-job training and technology upgrade courses utilizing modern information technologies like internet platforms to enhance employees’ professional skills and service efficiency; promoting the concept of lifelong learning, establishing special funds or tax incentives encourages enterprises and individuals to participate in continuing education programs to improve employees’ knowledge levels and vocational skills, enhancing their competitiveness in the workplace and personal sense of achievement.

Given China’s unique labor market and socioeconomic conditions, the results of this study have typicality. Although the results of this study are based on data from Chinese employees, they are applicable to some extent to other developing economies. China’s rapid industrialization and urbanization have led to a transition from agriculture to industry and service sector dominance, resulting in a series of job quality-related issues such as poor working environments, low wages, and inadequate social security systems. Meanwhile, Chinese culture emphasizes collectivism and individual responsibility towards the family, values that may influence people’s attitudes and expectations toward work, i.e., work is not only a source of personal income but also an important path to obtaining social status and family respect. Moreover, the Chinese government has introduced a series of policies and measures aimed at improving job quality and reducing inequality in recent years, directly impacting the well-being of workers.

While acknowledging these specificities, the results of this study possess a certain degree of universality and can be extended to other developing economies. Many developing countries face similar labor market segmentation issues, with significant disparities between formal and informal sectors, indicating that enhancing job quality is equally crucial for promoting workers’ happiness in these nations. Whether in Asian or Latin American developing countries, high-quality jobs often come with better health behaviors and stronger professional identity. Thus, the mechanism of improving employee health by enhancing job quality may be universal. As globalization accelerates and economic exchanges between countries become more frequent, the concepts of job quality and workers’ well-being have become mutually reinforcing, and many developing countries have begun to focus on the creation of decent work as a key component of achieving sustainable development goals.

## Data Availability

Publicly available datasets were analyzed in this study. This data can be found here: the data are available from the China Family Panel Studies (CFPS). This is an open source data where anyone can access through application with the Institute of Social Science Survey (ISSS) of Peking University (https://opendata.pku.edu.cn/, Email: opendata@lib.pku.edu.cn).
